# Estrogen-related receptor alpha induces epithelial-mesenchymal transition through cancer-stromal interactions in endometrial cancer

**DOI:** 10.1038/s41598-019-43261-z

**Published:** 2019-04-30

**Authors:** Kaori Yoriki, Taisuke Mori, Tetsuya Kokabu, Hiroshi Matsushima, Shiori Umemura, Yosuke Tarumi, Jo Kitawaki

**Affiliations:** Department of Obstetrics and Gynecology, Kyoto Prefectural University of Medicine, Graduate School of Medical Science, 465 Kajii-cho, Kawaramachi-Hirokoji, Kamigyo-ku, Kyoto, 602-8566 Japan

**Keywords:** Endometrial cancer, Cancer microenvironment, Endometrial cancer

## Abstract

Estrogen-related receptor alpha (ERRα), which shares structural similarities with estrogen receptors, is associated with tumor progression in endometrial cancer, but little is known about the detailed underlying mechanism. We investigated whether ERRα, in cooperation with peroxisome proliferator-activated receptor gamma coactivator 1-alpha (PGC-1α), could participate in epithelial-mesenchymal transition (EMT) in endometrial cancer through cancer-stromal interactions. Two endometrial cancer cell lines, Ishikawa and HEC-1A, transfected with ERRα/PGC-1α expression plasmids or silenced for ERRα expression, were co-cultured with telomerase-transformed human endometrial stromal cells (T-HESCs). We found that EMT-associated factors including vimentin, Snail, and zinc finger E-box binding homeobox 1 were upregulated in cancer cells overexpressing ERRα/PGC-1α and that transforming growth factor-beta (TGF-β) was induced in T-HESCs in the same conditions. In contrast, ERRα knockdown suppressed EMT-associated factors in cancer cells and TGF-β in T-HESCs. ERRα/PGC-1α overexpression increased the expression of EMT-associated factors after TGF-β exposure; however, it decreased E-cadherin at protein level. ERRα knockdown suppressed EMT-associated factors in the presence of TGF-β, whereas E-cadherin remained unchanged. Matrigel invasion assays revealed that ERRα knockdown attenuated the stimulation of migration and invasion by TGF-β. These findings suggest that ERRα is a potential target for inhibiting TGF-β-induced EMT through cancer-stromal interactions in endometrial cancer.

## Introduction

Endometrial cancer is a leading cause of female genital tract malignancies, and its incidence and death rates have been notably increasing. Most patients (75%) are diagnosed when the disease is still confined to the uterus (International Federation of Gynecology and Obstetrics [FIGO] stage I or II), with 5-year overall survival rates ranging from 74% to 91%^1^. However, for FIGO stage III and IV, the 5-year overall survival is 57–66% and 20–26%, respectively^1^. Although several treatment options such as surgery, hormonal therapy, radiation therapy, and chemotherapy are effective for localised disease, only limited options remain if the tumor metastasizes to extrauterine regions^2^. Therefore, it is important to understand the molecular mechanisms underlying the progression of the disease toward invasion and metastasis.

The conversion of early-stage into advanced-stage tumors is associated with activation of epithelial-mesenchymal transition (EMT), which plays a crucial role in cancer invasion and metastasis^3–6^. The acquisition of mesenchymal properties through EMT can promote the detachment of cancer cells from the primary tumor and facilitate their subsequent aggressive dissemination, allowing metastasis to occur^7^. The connection between the loss of E-cadherin-mediated cell-to-cell adhesion in cancer cells and EMT has been established previouly^8^. EMT involves the induction or functional activation of mesenchymal markers such as vimentin, and a series of EMT-inducing transcription factors, including Snail and zinc finger E-box binding homeobox 1 (ZEB1). The full spectrum of signaling pathways contributing to EMT in cancer cells remains unclear. Genetic and epigenetic alterations that occur in cancer cells during the course of the primary tumor formation render them especially responsive to EMT-inducing heterotypic signals originating in the tumor microenvironment^5^.

The tumor microenvironment includes the extracellular matrix (collagen and hyaluronic acid) and many secreted soluble factors, such as Wnt, transforming growth factor beta (TGF-β), Hedgehog, epidermal growth factor, hepatocyte growth factor, and cytokines^9,10^. Among these signals, TGF-β is the most potent inducer of EMT. TGF-β typically has tumor-suppressing activity in normal cells and early-stage cancers through its ability to induce cell cycle arrest and apoptosis. However, it can serve as a positive regulator of tumor progression and metastasis^5,11,12^. This switch in TGF-β function is known as the “TGF-β paradox” and is intimately linked to the initiation of EMT^13^.

Estrogen-related receptor alpha (ERRα), one of the orphan nuclear receptors, shows structural similarities with estrogen receptors, but does not bind to natural estrogens^14^. ERRα is expressed mostly in tissues with high metabolic demand such as the skeletal muscle, kidney, heart, liver, and adipose tissue^15^, and plays a predominant role in orchestrating mitochondrial biogenesis and cellular energy metabolism (e.g., oxidative phosphorylation, tricarboxylic acid cycle, fatty acid oxidation, ATP synthesis, and aerobic glycolysis)^16–18^. Peroxisome proliferator-activated receptor gamma coactivator 1-alpha (PGC-1α), a critical regulator of genes controlling many aspects of energy metabolism, is a powerful transcriptional coactivator of ERRα and regulates the expression and activity of ERRα^19,20^. ERRα has been reported to be elevated in multiple human cancers including breast, colorectal, ovary, and endometrial cancer^21–23^, and its expression is correlated with poor prognosis^24–26^. We previously demonstrated the tumor-promoting action of ERRα and its association with poor prognosis in endometrial cancer^27^. However, little has been reported on the detailed underlying mechanism. Here, we investigated whether ERRα could activate EMT of cancer cells through cancer-stromal interactions and thereby promote invasion and metastasis in endometrial cancer.

## Results

### Upregulation of ERRα in cancer cells induces ERRα and TGF-β in stromal cells

**Figure 1:**
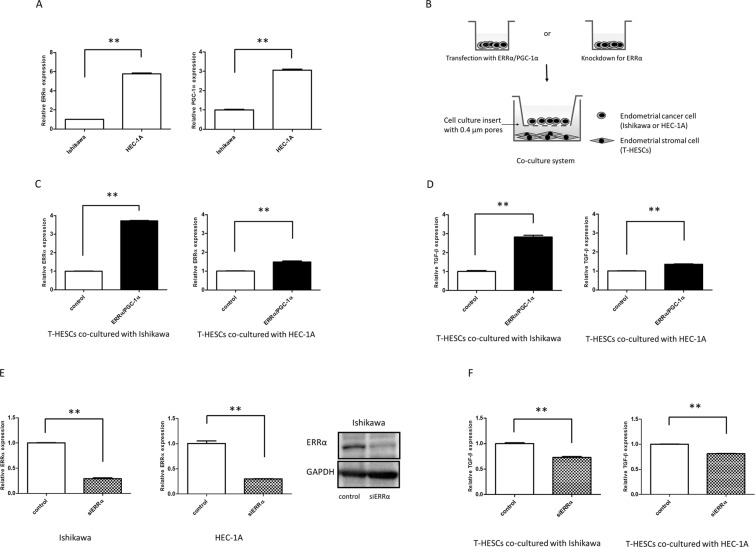
Effects of ERRα in cancer cells on ERRα and TGF-β in stromal cells. (**A**) The expression of ERRα and PGC-1α in Ishikawa and HEC-1A cells was examined by qPCR. (**B**) T-HESCs were co-cultured with Ishikawa or HEC-1A cells that had been transfected with ERRα/PGC-1α expression plasmids or silenced for ERRα expression. The expression of ERRα (**C**) and TGF-β (**D**) was measured using qPCR, in T-HESCs co-cultured with cancer cells overexpressing ERRα/PGC-1α. (**E**) The effect of ERRα knockdown was evaluated by qPCR and western blot analysis. (**F**) TGF-β expression was examined in T-HESCs co-cultured with cancer cells with ERRα knockdown. Data represent the mean ± standard error of the mean. *P* values are based on Student’s *t-*test. **P < 0.01.

We first confirmed that HEC-1A cells showed higher expression of ERRα and PGC-1α than Ishikawa cells (Fig. 1A). To investigate the cancer-stromal interactions through ERRα in endometrial cancer, human endometrial fibroblasts immortalized with human telomerase reverse transcriptase (T-HESCs) were co-cultured with Ishikawa or HEC-1A cells overexpressing ERRα/PGC-1α (Fig. 1B). Quantitative polymerase chain reaction (qPCR) assays revealed increased levels of ERRα in T-HESCs co-cultured with Ishikawa and HEC-1A cells overexpressing ERRα/PGC-1α (*P* < 0.01, Fig. 1C). TGF-β is an important mediator in cancer-stromal signaling. Our results showed that overexpression of ERRα/PGC-1α in both cancer cell types induced TGF-β expression in T-HESCs (*P* < 0.01, Fig. 1D).

We also performed loss-of-function experiments using siRNA (Fig. 1B). After ERRα knockdown was confirmed by qPCR (*P* < 0.01) and western blot analysis (Fig. 1E), we examined TGF-β levels in T-HESCs co-cultured with Ishikawa or HEC-1A cells. ERRα knockdown significantly suppressed TGF-β expression in T-HESCs (*P* < 0.01, Fig. 1F).

### ERRα/PGC-1α overexpression induces the expression of EMT markers and the motility of cancer cells through cancer-stromal interactions

**Figure 2:**
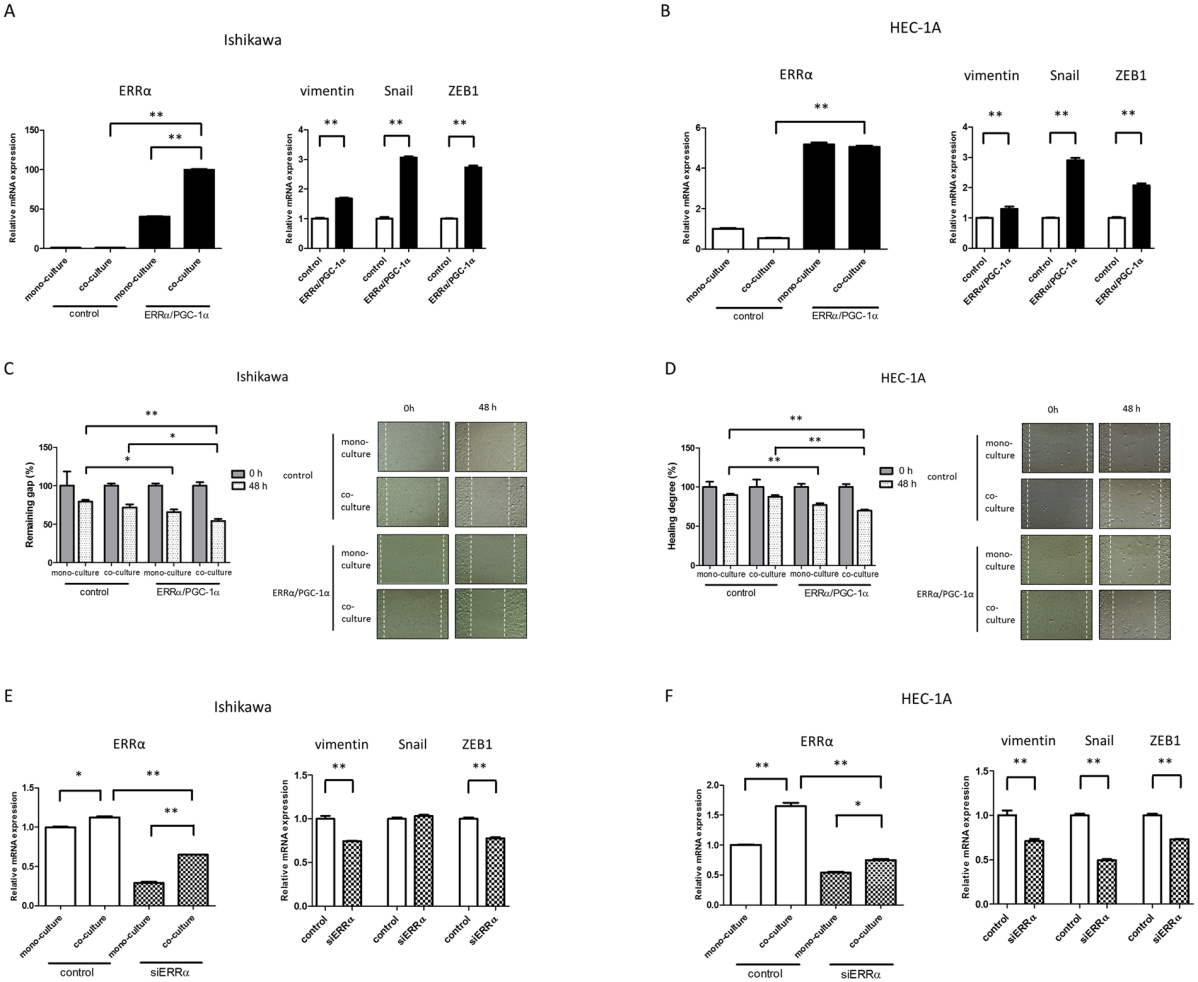
Effects of ERRα on EMT markers and motility of cancer cells through cancer-stromal interactions. The expression of vimentin, Snail, and ZEB1 in Ishikawa (**A**) and HEC-1A (**B**) cells co-cultured with T-HESCs, after overexpression of ERRα/PGC-1α, was examined by qPCR. The wound healing assays of Ishikawa (**C**) and HEC-1A (**D**) cells co-cultured with T-HESCs, after knockdown of ERRα (n = 3). The expression of vimentin, Snail, and ZEB1 in Ishikawa (**E**) and HEC-1A (**F**) cells co-cultured with T-HESCs, after ERRα knockdown, was examined by qPCR. Data represent the mean ± standard error of the mean. *P* values are based on Student’s *t*-test and one-way ANOVA with post-hoc Tukey’s multiple comparison test. *P < 0.05; **P < 0.01.

To elucidate the association between cancer-stromal interactions and ERRα, we next focused on EMT, as TGF-β is a critical modulator of EMT in tumor progression. The expression of vimentin, Snail, and ZEB1 in cancer cells co-cultured with T-HESCs was examined by qPCR. In Ishikawa and HEC-1A cells overexpressing ERRα/PGC-1α, vimentin, Snail, and ZEB1 were upregulated (*P* < 0.01, Fig. 2A,B). However, in the monolayer culture system, these responses were weak (data not shown). Wound healing assays showed that ERRα/PGC-1α overexpression significantly induced the motility of Ishikawa and HEC-1A cells (*P* < 0.05 and *P* < 0.01 in Ishikawa and HEC-1A cells, respectively). Furthermore, co-culture with T-HESCs tended to increase the responses (Fig. 2C,D). In contrast, vimentin and ZEB1 expression was reduced significantly in Ishikawa cells with silencing of ERRα (*P* < 0.01), although Snail expression did not change (Fig. 2E). Similarly, the expression of vimentin, Snail, and ZEB1 decreased significantly in HEC-1A cells in which ERRα was silenced (*P* < 0.01, Fig. 2F).

### Upregulation of ERRα is involved in TGF-β-induced EMT

**Figure 3:**
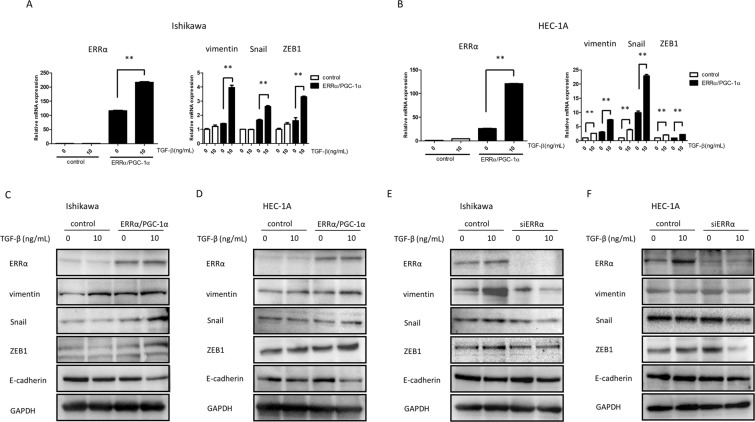
Effects of ERRα on TGF-β-induced EMT markers. The expression of ERRα, vimentin, Snail, and ZEB1 was examined in Ishikawa (**A**) and HEC-1A (**B**) cells mono-cultured in the presence of 10 ng/mL TGF-β, after overexpression of ERRα/PGC-1α. Western blot analysis of E-cadherin, vimentin, Snail, and ZEB1 in cancer cells treated with TGF-β, after overexpression of ERRα/PGC-1α (**C**,**D**). Western blot analysis of these EMT-associated factors in both cancer cell types with ERRα knockdown (E and F). Full-length blots are presented in Supplementary Figs 1–4. Data represent the mean ± standard error of the mean. *P* values are based on Student’s *t*-test. **P < 0.01.

To elucidate the effect of ERRα on TGF-β-induced EMT, we treated Ishikawa and HEC-1A cells overexpressing ERRα/PGC-1α with TGF-β in a monoculture system. Interestingly, ERRα/PGC-1α overexpression significantly induced the expression of vimentin, Snail, and ZEB1 in Ishikawa and HEC-1A cells treated with 10 ng/mL TGF-β (*P* < 0.01), whereas TGF-β exposure did not have this effect in parental Ishikawa cells (Fig. 3A) and its effect was very limited in parental HEC-1A cells (Fig. 3B). To further confirm this observation, we used western blot analyses to assess the expression of EMT-associated factors. ERRα/PGC-1α overexpression stimulated the expression of vimentin, Snail, and ZEB1 after exposure to TGF-β and reduced the expression of E-cadherin in Ishikawa and HEC-1A cells (Fig. 3C,D). ERRα knockdown reduced the expression of vimentin, Snail, and ZEB1 in the presence of TGF-β. No changes in E-cadherin expression were noted upon ERRα silencing in both cancer cell types (Fig. 3E,F).

### ERRα knockdown suppresses TGF-β-induced migration and invasion in endometrial cancer cells

**Figure 4:**
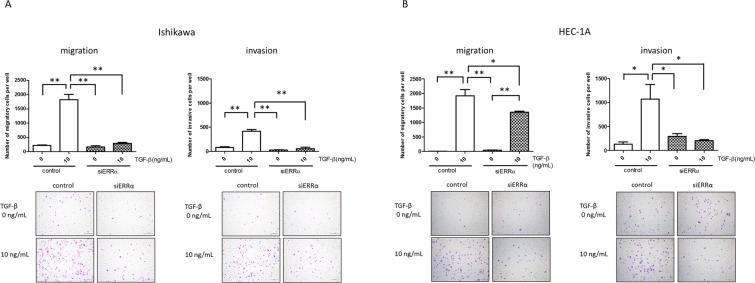
Effects of ERRα knockdown on TGF-β-induced migration and invasion. The migratory and invasive capabilities in Ishikawa and HEC-1A cells treated with TGF-β, after ERRα knockdown, were evaluated using trans-well cell culture and Matrigel invasion chamber systems. Data represent the mean ± standard error of the mean (Ishikawa cells, n = 2; HEC-1A cells, n = 3; **A**,**B**). *P* values are based on one-way ANOVA with post-hoc Tukey’s multiple comparison test. *P < 0.05; **P < 0.01.

Finally, we evaluated the effect of ERRα on the TGF-β-induced migration and invasion of Ishikawa and HEC-1A cells, using trans-well cell culture and Matrigel invasion chamber systems. We first confirmed that TGF-β exposure significantly induced migration and invasion of Ishikawa and HEC-1A cells (*P* < 0.01, Fig. 4A,B). We next assessed whether ERRα could regulate migration and invasion in these cells and found that ERRα knockdown attenuated the stimulation of migration and invasion by TGF-β (*P* < 0.01 and *P* < 0.05 in Ishikawa and HEC-1A cells, respectively, Fig. 4A,B).

## Discussion

We elucidated the influence of the tumor microenvironment on the establishment and maintenance of EMT in endometrial cancer cells by co-culturing human endometrial cancer cells and a human endometrial fibroblast cell line. The co-culture system is a useful approach to mimic cancer-stromal interactions. TGF-β plays a key role in tumor progression and metastasis in the tumor microenvironment. Increased expression of TGF-β in various cancers is highly correlated with poor prognosis^28,29^. However, the function of TGF-β in endometrial cancer remains unclear. Our study focused on determining the effect of TGF-β signaling on the cancer-stromal interactions in endometrial cancer.

First, our results indicated that upregulation of ERRα in endometrial cancer cells induces TGF-β and ERRα expression in stromal cells. A previous study identified soluble factors produced by fibroblasts that stimulate invasion of cancer cells using a co-culture model with breast cancer cell lines and fibroblast cell line established from healthy mammary tissue^30^. Similarly, in this study, we used an endometrial fibroblast cell line rather than primary fibroblasts isolated from cancer tissues as a non-malignant stromal cell population surrounding the cancer cells. This allowed us to investigate the influence of the altered expression of ERRα in cancer cells on stromal cells. Accumulating evidence suggests that cancer-associated fibroblasts (CAFs) originate from resident tissue fibroblasts^31–33^. CAFs are the main contributors to the association between stromal TGF-β-driven programs and poor clinical outcome in colorectal cancer^34^. Furthermore, the interaction of colon cancer cells with resident fibroblasts hyperactivates TGF-β signaling in fibroblasts^35^. Our data indicated that TGF-β signaling in endometrial stromal cells may be mediated by the paracrine ERRα signals of endometrial cancer cells through cancer-stromal interactions.

Second, our results demonstrated that ERRα induces the expression of EMT markers in cancer cells through cancer-stromal interactions. Several studies have described the prognostic impact of the expression of EMT-related factors in endometrial cancer^36,37^. The increased expression of Snail and ZEB1 is closely related to decreased E-cadherin expression^38,39^, and this pattern is significantly correlated with myometrial invasion, histological type, and patients’ decreased survival^40,41^. Our data also revealed that cancer-stromal interactions may promote the motility of cancer cells overexpressing ERRα. Some reports have shown that the interaction between CAFs and cancer cells could enhance the metastatic potential of cancer cells through EMT induced by paracrine TGF-β signaling^42,43^. Additionally, co-cultures of squamous cell carcinoma cells and stromal fibroblasts revealed that cancer cells moved within tracks in the extracellular matrix produced by the fibroblasts^44^. In line with these data, our study indicated that stromal cells contribute to EMT in endometrial cancer via secretion of TGF-β.

Finally, our results demonstrated that ERRα promotes the migration and invasion of endometrial cancer cells by enhancing the TGF-β-induced EMT. In support of our results, previous studies have shown that ERRα promotes the migration and invasion of cancer cells by inducing EMT in some kinds of cancers^45–48^. Huang X *et al*. recently reported that ERRα directly binds to the promoter of TGF-β and increases the positive self-feedback regulation of TGF-β in endometrial cancer cells^48^. Our data indicated that the upregulation of ERRα in cancer cells induces TGF-β expression in stromal cells and the exogenous TGF-β signaling influences EMT in cancer cells. Our findings from the co-culture system suggest that exogenous TGF-β signaling through the cancer-stromal interactions might be more important than endogenous TGF-β from only epithelial cancer cells. Chen Y *et al*. reported that ERRα could mediate the TGF-β-induced EMT via the transcription of Snail in osteosarcoma cells^47^. Our study demonstrated that ZEB1 and Snail participate in the ERRα-mediated EMT stimulated by TGF-β in endometrial cancer. Our finding does provide further insight into the potential role of ERRα in progression of endometrial cancer. Furthermore, the level of ERRα in endometrial cancer cells was further increased by positive feedback through TGF-β. In rapidly growing cancers, the relative shortage of blood flow causes ischemia and hypoxia, which increase the expression of ERRα/PGC-1α in cancer cells. We and other investigators have previously demonstrated that ERRα/PGC-1α promotes tumor angiogenesis via induction of VEGF transcription^27,49^. Therefore, ERRα may be involved in both tumor angiogenesis and tumor invasion in the advanced stages of cancer.

There are several limitations to this study. Although we evaluated EMT *in vitro* based on downregulation of E-cadherin and upregulation of vimentin, we have not evaluated EMT of cancer cells *in vivo*. The cancer stroma *in vivo* has many components other than fibroblasts, such as the extracellular matrix, vascular endothelial cells, immune cells and adipocytes^50,51^. Moreover, we did not consider how these components function in the tumor microenvironment.

In conclusion, our study revealed for the first time that ERRα plays a crucial role in the TGF-β-induced EMT through cancer-stromal interactions in endometrial cancer cells. Growing evidence indicates targeting TGF-β signaling for cancer treatment is difficult, as TGF-β regulates a broad range of cellular responses, including cell proliferation, differentiation, and apoptosis. Targeting ERRα may be an effective alternative approach for endometrial cancer treatment.

## Methods

### Cell lines and culture

The human uterine endometrial cancer cell line Ishikawa was provided by the Cell Resource Center for Biomedical Research (Institute of Development, Aging and Cancer, Tohoku University, Sendai, Japan). The human uterine endometrial cancer cell line HEC-1A and the telomerase-transformed human endometrial stromal cells T-HESCs were purchased from the American Type Culture Collection (Manassas, VA, USA). Ishikawa and HEC-1A cells were maintained in Minimum Essential Medium (MEM) (Nacalai Tesque, Kyoto, Japan) with sodium pyruvate, and T-HESCs were cultured in Dulbecco’s Modified Eagle’s Medium/Ham’s F-12 (Nacalai Tesque). Each medium was supplemented with 10% fetal bovine serum (FBS) (Biowest, Nuaille, France) and penicillin-streptomycin (Nacalai Tesque). All cells were cultured at 37 °C in a humidified 5% CO_2_ atmosphere.

TGF-β was purchased from R&B systems (Minneapolis, MN, USA). Ishikawa and HEC-1A cells were incubated in phenol-red free MEM supplemented with 10 ng/mL TGF-β in a humidified 5% CO_2_ atmosphere for 48–72 h.

### Co-culture system

For physical separation of the endometrial cancer cell lines and T-HESCs, transwell cultures were established in 6- or 12-well plates using Falcon Cell Culture Inserts (pore size 0.4 μm) and Cell Culture Companion Plates (Corning, Corning, NY, USA). First, Ishikawa cells, HEC-1A cells, and T-HESCs were cultured separately until sub-confluency in separate culture plates. T-HESCs were seeded at 1 × 10^5^ cells/mL in Cell Culture Companion Plates, and on the next day, 1 × 10^5^ cells/mL Ishikawa or HEC-1A cells were seeded in the Cell Culture Inserts placed on the top of the T-HESCs. After 24 h of co-culture, Ishikawa or HEC-1A cells and T-HESCs were separated again and examined by qPCR.

### Plasmids

The ERRα and PGC-1α expression plasmids were constructed by inserting the full-length human *ERRα* and *PGC-1α* genes (NM_004451 and NM_013261) into the pSG5-empty vector (Promega, Madison, WI, USA) to obtain pSG5-ERRα and pSG5-PGC-1α and were kindly provided by Prof. Shiuan Chen. The pSG5-empty plasmid (Promega) was used as a control.

### Transient transfection

The transfection experiments were performed on subconfluent cells. Plasmids (pSG5-empty, pSG5-ERRα, and pSG5-PGC-1α) were transfected using Lipofectamine LTX (Invitrogen, Carlsbad, CA, USA) according to the manufacturer’s protocol. After a 4-h incubation, the transfection medium, containing Lipofectamine LTX and plasmids was removed and replaced with phenol red-free MEM supplemented with 10% dextran-coated charcoal-treated FBS (Biowest) and antibiotics. After a 48-h incubation, the cells were processed for analysis.

### RNA interference

The small interfering RNA (siRNA) for ERRα (s4831) and the negative control siRNA (control #1) were Silencer Select siRNAs purchased from Ambion (Austin, TX, USA). The siRNA transfection experiments were performed using Lipofectamine RNAiMAX (Invitrogen) according to the manufacturer’s protocol. We confirmed the knockdown efficiency of the siRNAs at a final concentration of 10 nM using qPCR and western blotting analysis. The negative control siRNA was used as a control.

### RNA extraction and qPCR

RNA extraction and qPCR were performed as previously described^52^. Total RNA (1 μg) was extracted from cultured cells using the RNeasy Mini kit (QIAGEN, Venlo, Netherlands), and then cDNA was synthesized from 1 μg of RNA using the ReverTra Ace qPCR RT kit (Toyobo, Osaka, Japan). qPCR was performed using the CFX Connect Real-time PCR Detection System (Bio-Rad, Hercules, CA, USA). cDNA samples generated from the total RNA from cells (1 μL) were mixed into 20-μL reactions containing SYBR qPCR Thunderbird master mix (Toyobo) and each primer at 0.3 μM. The following primers were designed with the Primer3Plus free software and purchased from Invitrogen: ERRα 5′-GGCCCTTGCCAATTCAGA-3′ (forward) and 5′-GGCCTCGTGCAGAGCTTCT-3′ (reverse), PGC-1α 5′-TGTGGATGAAGACGGATTGC-3′ (forward) and 5′-GTCAGGCATGGAGGAAGGAC-3′ (reverse), TGF-β 5′-CACTCCCACTCCCTCTCTC-3′ (forward) and 5′-GTCCCCTGTGCCTTGATG-3′ (reverse), E-cadherin 5′-GAGGAGAGcGGTGGTCAAAG-3′ (forward) and 5′-TCCGCCTCCTTCTTCATCAT-3′ (reverse), vimentin 5′-TTGCAGGAGGAGATGCTTCA-3′ (forward) and 5′-GTCAAGACGTGCCAGAGACG-3′ (reverse), Snail 5′-GGCTCCTTCGTCCTTCTCCT-3′ (forward) and 5′-CTGGAGATCCTTGGCCTCAG-3′ (reverse), ZEB1 5′-CACTGCCCAGTTACCCACAA-3′ (forward) and 5′-GGGGAATCAGAATCGTTTGC-3′ (reverse), and glyceraldehyde 3-phosphate dehydrogenase (GAPDH) 5′-GCACCGTCAAGGCTGAGAAC-3′ (forward) and 5′-ATGGTGGTGAAGACGCCAGT-3′ (reverse). The mRNA levels of the target genes were quantified using the comparative method (∆∆CT method) and normalized to GAPDH expression.

### Wound healing assay

Ishikawa (3.0 × 10^5^) or HEC-1A (3.3 × 10^5^) cells were seeded in the Cell Culture Inserts and T-HESCs (3.0 × 10^5^) were seeded in Cell Culture Companion Plates. On the next day, Ishikawa or HEC-1A cells were wounded with 200-μL pipette tip and placed on the top of T-HESCs or only medium. After a 48-h incubation, the distance migrated was quantitated by acquiring images. Photomicrographs were acquired using a Keyence BZ-X700 microscope (Keyence, Osaka, Japan).

### Migration and invasion assay

Migration and invasion assays were performed using uncoated and Matrigel-coated double-chamber systems (pore size 8 μm, Corning BioCoat Matrigel Invasion Chamber, Corning) as previously described^52^. Ishikawa (4.5 × 10^4^) or HEC-1A (5.0 × 10^4^) cells were seeded into the upper chamber (uncoated or Matrigel-coated inserts) filled with serum-free medium; 10% FBS-containing medium was used as a chemoattractant in the lower chamber. After 24 h, the cells that migrated and invaded into the lower side of the inserts were fixed and stained with the Diff-Quik kit (Sysmex, Kobe, Japan). The number of stained cells was counted in the whole field using an Olympus BX50 microscope (Olympus, Tokyo, Japan) (200×), and photomicrographs were acquired using an Olympus DP22 (Olympus) (100×).

### Antibodies

The mouse anti-ERRα (sc-65715), anti-vimentin (sc-66002), and anti-SNAI1 (sc-271977) antibodies were purchased from Santa Cruz Biotechnology (Dallas, TX, USA). The mouse anti-E-cadherin antibody (M106) was purchased from Takara Bio Inc. (Shiga, Japan). The rabbit anti-SNAI1 antibody (SAB2108482) was purchased from Sigma-Aldrich (St. Louis, MO, USA). The rabbit anti-ZEB1 antibody (GTX105278) was purchased from Gene Tex (Los Angeles, CA, USA). The rabbit anti-GAPDH (#2118) antibody and the anti-rabbit (#7074) and anti-mouse (#7076) IgG, HRP-linked antibodies were obtained from Cell Signaling Technology (Beverly, MA, USA). All antibodies were used at the concentration recommended by the manufacturers.

### Western blot analysis

Western blot analysis was performed as previously described^52^. Cells were washed three times with phosphate-buffered saline and lysed in radioimmunoprecipitation assay (RIPA) buffer (Nacalai Tesque). Cell lysates (10–20 μg) were heated in sodium dodecyl sulfate (SDS) sample buffer (125 mM Tris-HCl, pH 6.8, 4% SDS, 25% glycerol, 10% 2-mercaptoethanol, 0.05 mM phenylmethanesulfonyl fluoride and 0.004% bromophenol blue), separated by 10% e-PAGEL (Atto Corp, Tokyo, Japan) according to the manufacturer’s recommendations, and transferred onto immuno-blot polyvinylidene fluoride (PVDF) membranes (Bio-Rad). The membranes were blocked in PVDF Blocking Reagent for Can Get Signal (Toyobo) for 1 h at 20–25 °C and incubated with the appropriate primary antibody in Can Get Signal Solution 1 (Toyobo) overnight at 4 °C. After washing, the membranes were incubated with the secondary antibody for 1 h at 20–25 °C. The signal was visualized by Chemi-Lumi One (Nacalai Tesque) and analyzed by ChemiDoc XRS + system with Image Lab software (Bio-Rad).

### Statistical analysis

Comparisons of the mean and standard error between two groups were made using two-tailed unpaired Student’s *t*-test. Comparisons of over 3 groups were made using one-way ANOVA with post-hoc Tukey’s multiple comparison test. These statistical analyses were performed using GraphPad Prism ver. 5.04 (GraphPad Software, San Diego, CA, USA). *P* < 0.05 was considered statistically significant.

## Supplementary information

Supplementary information

## Data Availability

The datasets generated and analyzed during the current study are available from the corresponding author on reasonable request.
