# Supplementary material for: Estrogen-related receptor alpha induces epithelial-mesenchymal transition through cancer-stromal interactions in endometrial cancer
Source: Sci Rep. 2019 Apr 30;9:6697. doi: 10.1038/s41598-019-43261-z (PMC6491648; doi:10.1038/s41598-019-43261-z)
Supplement: Supplementary file 1 — Supplementary information [file 41598_2019_43261_MOESM1_ESM.docx]

**Supplementary Information**

**Estrogen-related receptor alpha induces epithelial-mesenchymal transition through cancer-stromal interactions in endometrial cancer**

**Authors:** Kaori Yoriki, Taisuke Mori, Tetsuya Kokabu, Hiroshi Matsushima, Shiori Umemura, Yosuke Tarumi, Jo Kitawaki

**Supplementary Figure 1**

**
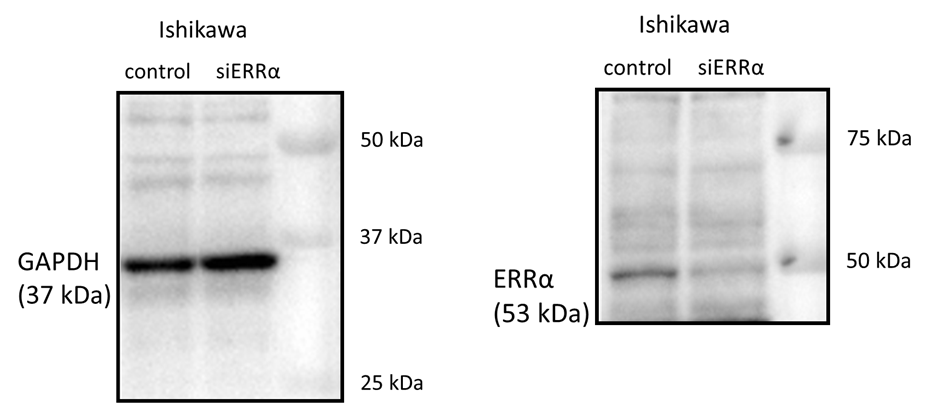
**

**Supplementary Fig. 1:** Full-length blots of ERRα and GAPDH protein expression for Figure 1E.

**Supplementary Figure 2**


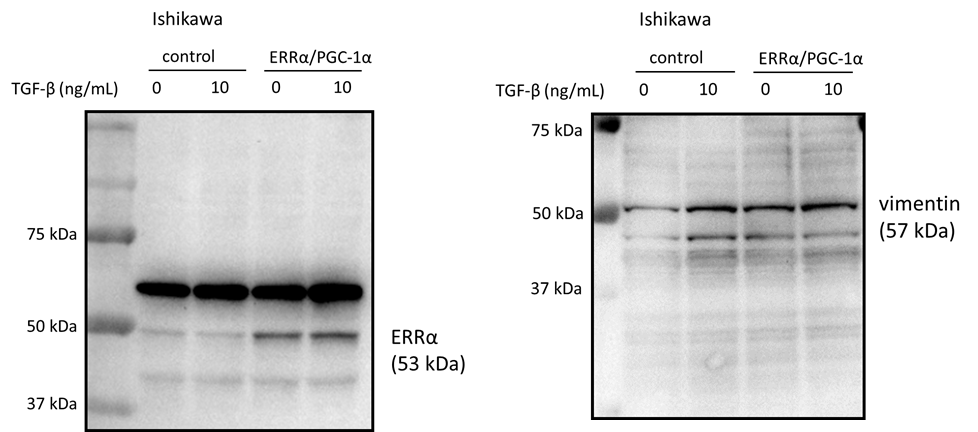


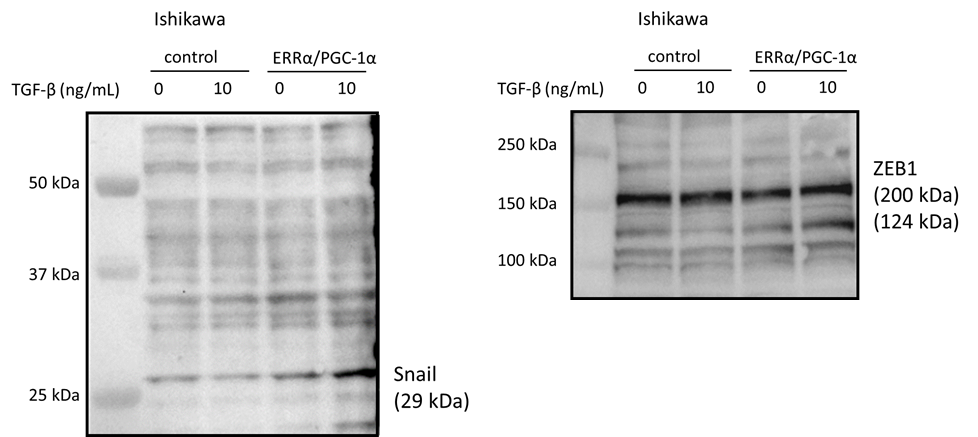


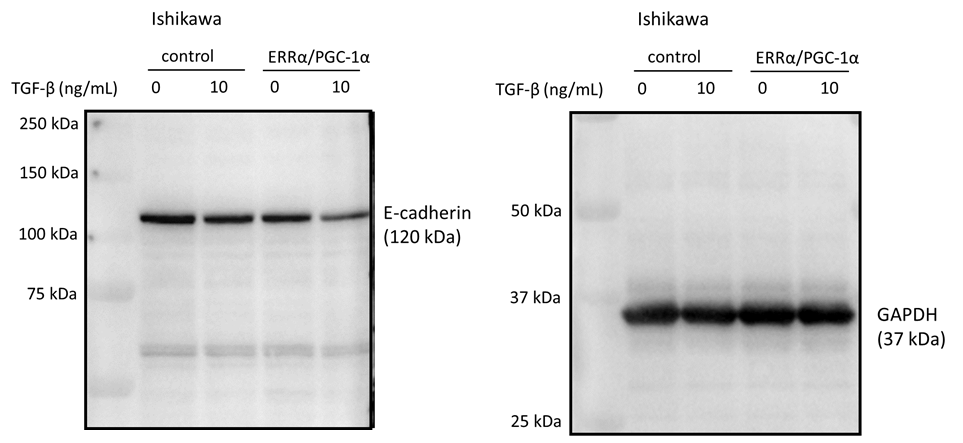


**Supplementary Fig. 2:** Full-length blots of ERRα, vimentin, Snail, ZEB1, E-cadherin, and GAPDH protein expression for Figure 3C.

**Supplementary Figure 3**


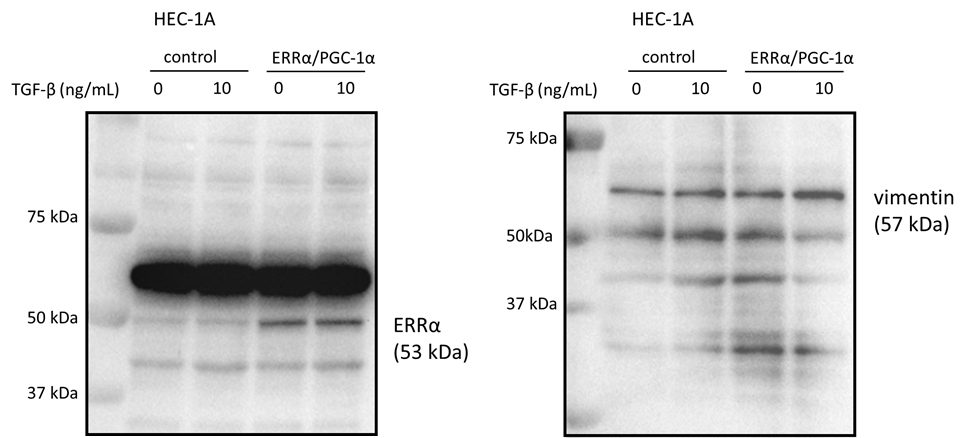


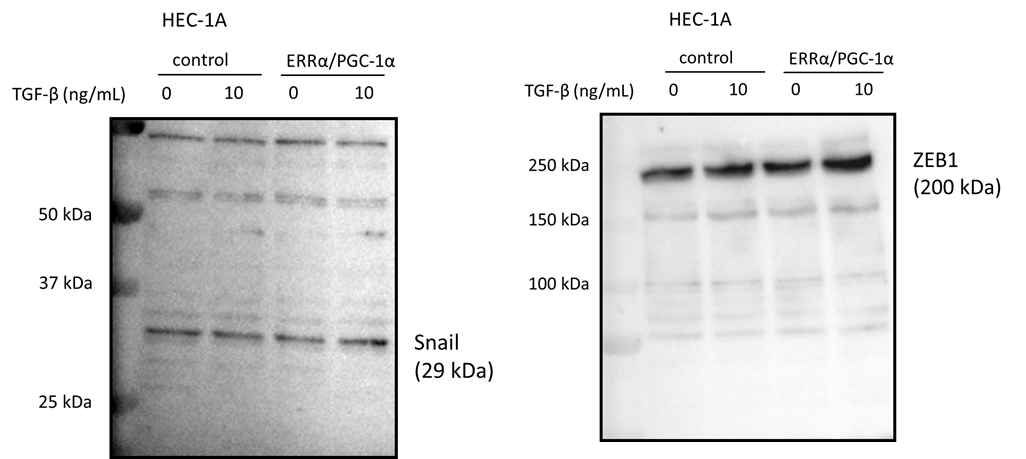


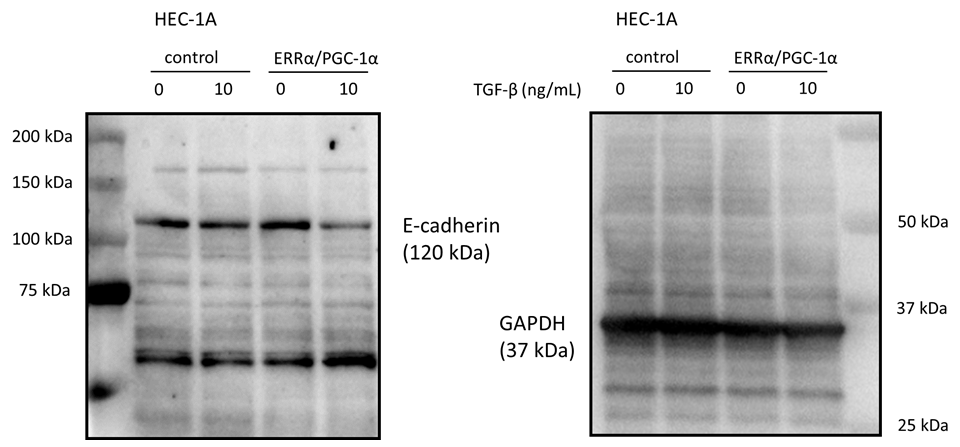


**Supplementary Fig. 3:** Full-length blots of ERRα, vimentin, Snail, ZEB1, E-cadherin, and GAPDH protein expression for Figure 3D.

**Supplementary Figure 4**


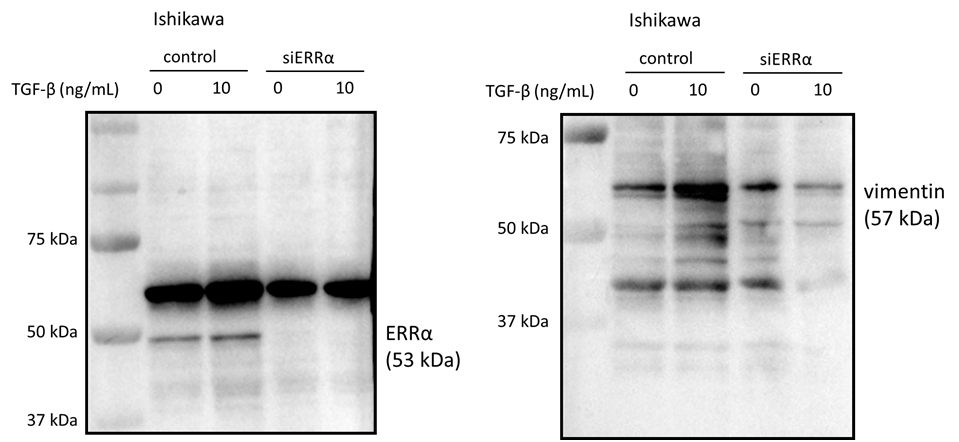


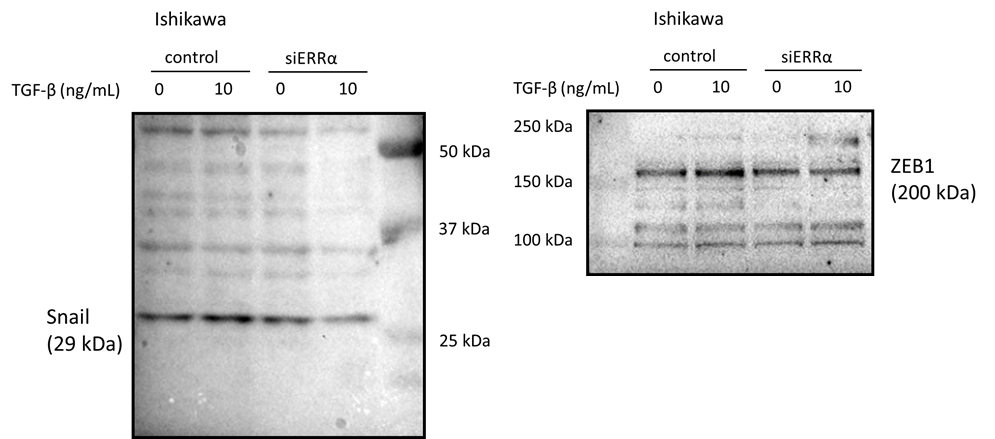


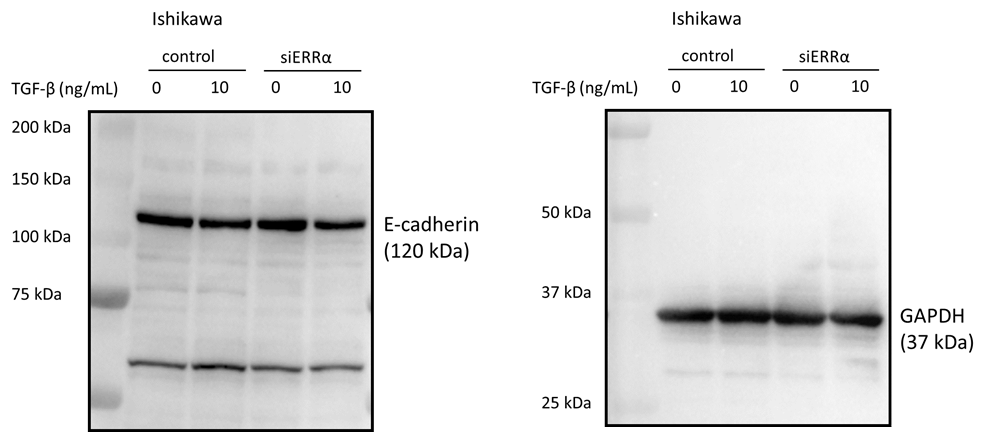


**Supplementary Fig. 4:** Full-length blots of ERRα, vimentin, Snail, ZEB1, E-cadherin, and GAPDH protein expression for Figure 3E.

**Supplementary Figure 5**


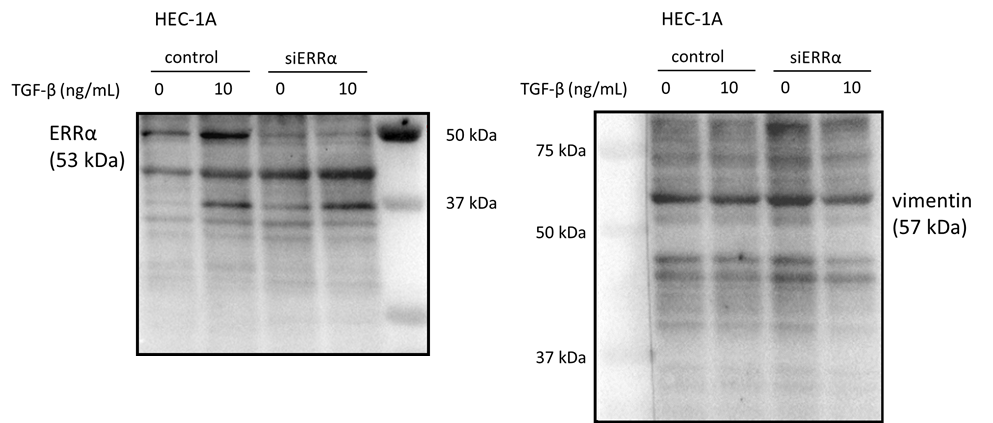


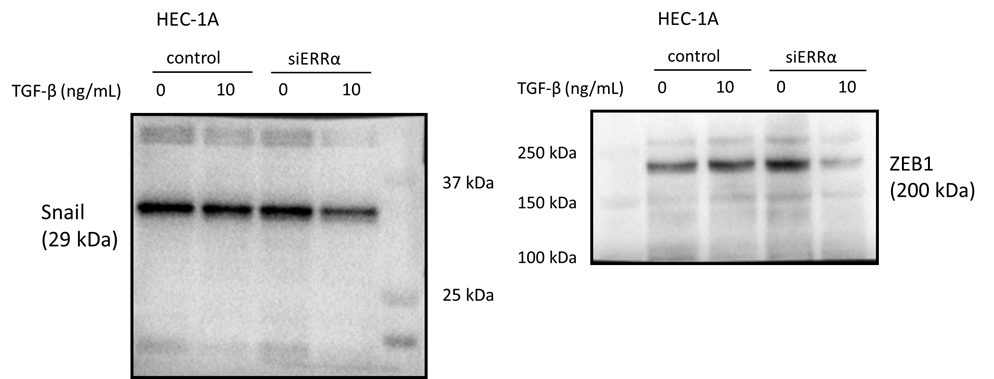


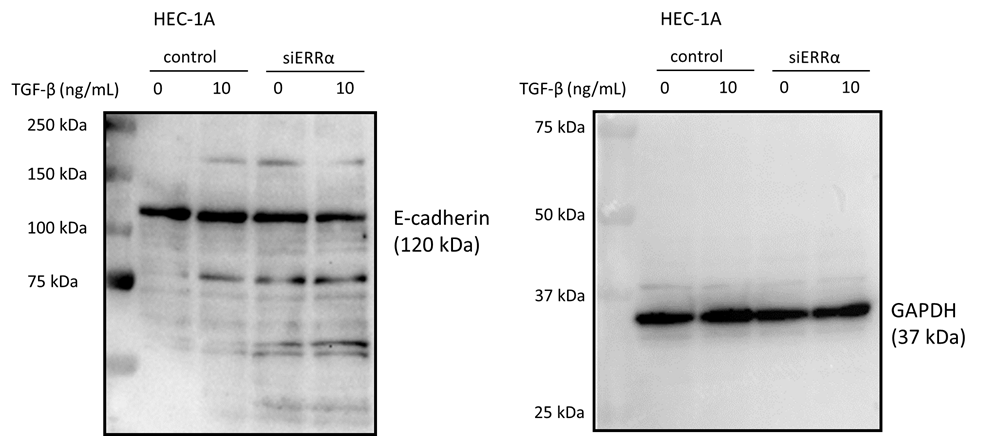


**Supplementary Fig. 5:** Full-length blots of ERRα, vimentin, Snail, ZEB1, E-cadherin, and GAPDH protein expression for Figure 3F.
